# Chromosomal organization of the 18S and 5S rRNAs and histone H3 genes in Scarabaeinae coleopterans: insights into the evolutionary dynamics of multigene families and heterochromatin

**DOI:** 10.1186/1471-2156-12-88

**Published:** 2011-10-15

**Authors:** Diogo C Cabral-de-Mello, Sárah G Oliveira, Rita C de Moura, Cesar Martins

**Affiliations:** 1 UNESP - Univ Estadual Paulista, Instituto de Biociências/IB, Departamento de Biologia, Rio Claro, São Paulo, Brazil; 2 UNESP - Univ Estadual Paulista, Instituto de Biociências/IB, Departamento de Morfologia, Botucatu, São Paulo, Brazil; 3 UPE - Univ de Pernambuco, Instituto de Ciências Biológicas/ICB, Departamento de Biologia, Recife, Pernambuco, Brazil

## Abstract

**Background:**

Scarabaeinae beetles show a high level of macro-chromosomal variability, although the karyotypic organization of heterochromatin and multigene families (rDNAs and histone genes) is poorly understood in this group. To better understand the chromosomal organization and evolution in this group, we analyzed the karyotypes, heterochromatin distribution and chromosomal locations of the rRNAs and histone H3 genes in beetles belonging to eight tribes from the Scarabaeinae subfamily (Coleoptera, Scarabaeidae).

**Results:**

The number of 18S rRNA gene (a member of the 45S rDNA unit) sites varied from one to 16 and were located on the autosomes, sex chromosomes or both, although two clusters were most common. Comparison of the 45S rDNA cluster number and the diploid numbers revealed a low correlation value. However, a comparison between the number of 45S rDNA sites per genome and the quantity of heterochromatin revealed (i) species presenting heterochromatin restricted to the centromeric/pericentromeric region that contained few rDNA sites and (ii) species with a high quantity of heterochromatin and a higher number of rDNA sites. In contrast to the high variability for heterochromatin and 45S rDNA cluster, the presence of two clusters (one bivalent cluster) co-located on autosomal chromosomes with the 5S rRNA and histone H3 genes was highly conserved.

**Conclusions:**

Our results indicate that the variability of the 45S rDNA chromosomal clusters is not associated with macro-chromosomal rearrangements but are instead related to the spread of heterochromatin. The data obtained also indicate that both heterochromatin and the 45S rDNA loci could be constrained by similar evolutionary forces regulating spreading in the distinct Scarabaeinae subfamily lineages. For the 5S rRNA and the histone H3 genes, a similar chromosomal organization could be attributed to their association/co-localization in the Scarabaeinae karyotypes. These data provide evidence that different evolutionary forces act at the heterochromatin and the 45S rDNA loci compared to the 5S rRNA and histone H3 genes during the evolution of the Scarabainae karyotypes.

## Background

Repetitive DNA elements constitute a large portion of eukaryote genomes and include satellites, minisatellites, microsatellites, transposable elements and some multigene families with high copy numbers [1,2]. Among them, ribosomal RNAs (rRNA) and histone genes are grouped into distinct multigene families that are organized in tandem with hundreds to thousands of copies of each [3,4]. The major ribosomal DNA cluster (tandem arrayed 45S rDNA repeating units) encodes for the 28S, 18S and 5.8S rRNAs, while the minor rDNA cluster (tandemly arrayed 5S rDNA repeating units) is responsible for the transcription of 5S rRNA [3]. The histone genes may be clustered into distinct chromosomal regions, and among invertebrates, these genes are typically clustered as quartets (H2A, H2B, H3, and H4) or quintets (H2A, H2B, H3, and H4 plus H1), although scattered solitary genes have also been reported [4-6].

Ribosomal RNA and multigene histone families are very useful cytogenetic markers for studying chromosomal diversification and genome organization. In insects, these sequences have been cytogenetically mapped more frequently in species belonging to the orders Coleoptera, Lepidoptera and Orthoptera, although studies are still incipient and have concentrated on mapping the major rDNA locus [7-11]. The mapping of other multigene families, such as the 5S rDNA locus and the histone genes, was primarily performed in specific groups, e.g., Acrididae [9,12] and Proscopiidae grasshoppers [10], chironomid midges [13] and fruit flies [14,15].

Scarabaeidae beetles comprise more than 25,000 species that are distributed worldwide and represent the largest group of the Scarabaeiodea superfamily [16,17]. Notably, the macro karyotypic structure of the Scarabaidae family is highly diverse in the representatives of subfamily Scarabaeinae (dung beetles), with variations in the diploid number that range from 2n = 8 to 2n = 24, distinct sex chromosome systems and chromosomal morphologies [18]. Scarabaeidae have been poorly studied with regards to chromosomal mapping of DNA sequences, and the analyses were primarily performed to describe the major rDNA clusters [19-27]. Only species from the genus *Dichotomius *have been analyzed with regard to the histone genes and the 5S rDNA locus [28].

Due to the high chromosomal diversity of Scarabaeinae beetles, the chromosomal organization and evolution of the group, the diploid number, the distribution of heterochromatin, the location of the 18S and 5S rRNA loci, and the histone H3 gene arrays were analyzed in several species that belong to different lineages, in order to understand the patterns of karyotypic evolution in the group. Our results showed distinct evolutionary patterns for the multigene families studied. The 5S rRNA and histone H3 gene clusters were primarily associated and were highly conserved in number, while the major rDNA locus and heterochromatin regions showed an intense turnover in their number and location in the Scarabaeinae karyotypes. These results are also discussed in the light of the possible mechanisms that are involved in diversification of repeated DNA elements.

## Results

### Karyotypes and heterochromatin distribution

Chromosomal complements and heterochromatin distribution patterns of 13 Scarabaeinae species were determined, including new descriptions and re-descriptions of previously published data (Table 1). The variation in the diploid number ranged from 2n = 8 to 2n = 20. The species were classified in three distinct groups of karyotypes based on their heterochromatin distribution pattern: (i) heterochromatin restricted to the centromeric/pericentromeric regions (non-spread pattern), (ii) heterochromatin in the centromeric/pericentromeric regions with additional terminal or subterminal heterochromatic blocks (moderately spread pattern), and (iii) high amount of heterochromatin, primarily represented by diphasic chromosomes (with heterochromatin blocks occupying one entire chromosomal arm) and large paracentromeric blocks of heterochromatin (highly spread pattern) (Table 1).

**Table 1:** Diploid numbers, heterochromatin patterns and chromosome location of rDNA clusters and H3 histone gene in 31 Scarabaeinae species

Tribe	Chromosomal formula (males)	Overall heterochromatin distribution	45S rDNA		5S rDNA		H3 histone		References
				
*Species*			Aut	Sex	Aut	Sex	Aut	Sex	
Ateuchini									
*Atheuchus *sp.	16 = 7 + Xy	Pericentromeric blocks	4	-	2	-			This work

Canthonini									
*Canthon staigi*	18 = 8 + Xy_p_	Pericentromeric blocks	2	-	2	-			This work
*Deltochilum calcaratum*	14 = 6 + neoXY	Pericentromeric blocks and diphasic chromosomes	4/6*	X/X, Y*	2	-			[26]; This work
*D. elevatum*	20 = 9 + Xy_p_		2	-	2	-			This work
*D. morbillosum*	14 = 6 + neoXY	Pericentromeric blocks and diphasic chromosomes	4	X					[26]
*D. verruciferum*	20 = 9 +XY_p_	Pericentromeric blocks and diphasic chromosomes	4	Y	2	-	2	-	This work

Coprini									
*Dichotomius affinis*	18 = 8 + Xy_p_		2	-	2	-			[28]
*D. bos*	18 = 8 + Xy_p_	Pericentromeric blocks	2	-	2	-	2	-	[28]
*D. crinicollis*	18 = 8 + Xy_p_	Pericentromeric blocks	2	X	2	-	2	-	[28]
*D. depresicollis*	18 = 8 + Xy_p_	Pericentromeric blocks	2	-	2	-	2	-	[28]
*D. geminatus*	18 = 8 +Xy_p_	Pericentromeric and terminal blocks	4	-	2	-	2	-	[28,63]
*D. laevicollis*	18 = 8 + Xy_p_	Pericentromeric blocks	2	-	2	-	2	-	[28]
*D. mormon*	18 = 8 + Xy_p_		3/4*	X/X*	2	-			[28]
*D*. aff *mundus*	18 = 8 + Xy_p_		2	-	2	-			[28]
*D. nisus*	18 = 8 + Xy_p_	Pericentromeric blocks	-	X, Y	2	-	2	-	[25,28]
*D. semianeus*	18 = 8 + Xy_p_		-	X	2	-			[28]
*D. semisquamosus*	18 = 8 + Xy_p_	Pericentromeric blocks	2/2*	-/X*	2	-	2	-	[25,28]
*D. sericeus*	18 = 8 + Xy_p_	Pericentromeric blocks	2	-	2	-	2	-	[25,28]
*D*. aff *sericeus*	18 = 8 + Xy_p_		2	-	2	-			[28]
*Dichotomius *sp.	18 = 8 + Xy_p_		2	-	2	-			[28]
*Ontherus appendiculatus*	20 = 9 + Xy_p_	Pericentromeric and terminal blocks	2	-	2	-			This work
*O. sulcator*	20 = 9 + Xy_p_		7/8*	-					This work

Oniticellini									
*Eurysternus caribaeus*	8 = 3 + neoXY	Pericentromeric blocks	-	X, Y	-	X	-	X	[24]; This work

Gymnopleurini									
*Gymnopleurus sturmi*	20 = 9 +Xy	Pericentromeric and subterminal blocks	4/5*	-					[22]

Onitini									
*Bubas bison*	20 = 9 + XY	Pericentromeric and large terminal blocks	8						[23]

Onthophagini									
*Digitonthophagus gazella*	20 = 9 + Xy_p_/Xy/Xy_r_	Pericentromeric blocks	2	-	2	-			This work

Phanaeini									
*Coprophanaeus cyanescens*	20 = 9 + XY_p_	Diphasic chromosomes	5/4*	-/X*	2	-	2	-	[27]; This work
*C. ensifer*	20 = 9 + XY	Diphasic chromosomes	15/10*	X/- *	-	X, Y	-	X, Y	[27]; This work
*Diabroctis mimas*	20 = 9 + Xy_p_/Xy	Paracentromeric blocks and diphasic chromosomes	4/6*	X/X*	5	X	5	X	[21]; This work
*Isocopris inhiata*	18 = 8 + Xy_p_	Peri- and paracentromeric blocks and diphasic chromosomes	2	-					[21]
*Phanaeus splendidulus*	20 = 9 + Xy_p_		7	-	2	-	2	-	This work

The references provided in the table are related to results of heterochromatin distribution or chromosomal mapping of rDNAs and H3 histone genes.

* Indicates the presence of polymorphism, being the two conditions observed in the same species separated by (/); Empty boxes: not available data;

### Mapping of multigene families

**Figure 1:**
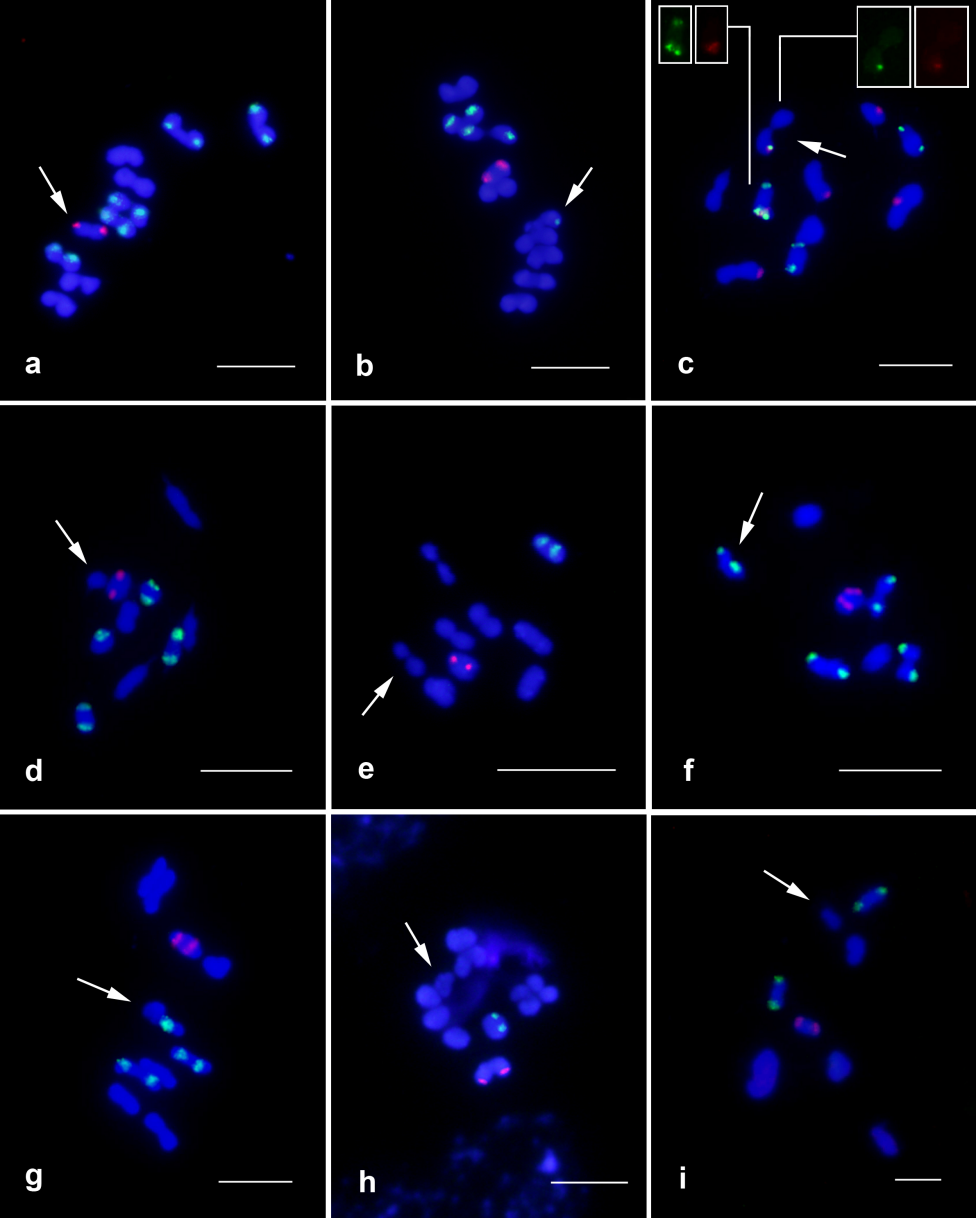
**Fluorescent *in situ *hybridization in metaphase I using 5S rDNA (red) and 18S rDNA (green) in nine representative species of Scarabaeinae that belong to three distinct tribes ([a-d] Phanaeini, [e-h] Canthonini, [i] Ateuchini)**. (a) *Coprophanaeus ensifer*, (b) *C. cyanescens*, (c) *Diabroctis mimas*, (d) *Phanaeus splendidulus*, (e) *Canthon staigi*, (f) *Deltochilum calcaratum*, (g) *D. verruciferum*, (h) *D. elevatum *and (i) *Atheuchus *sp. The arrows indicate the sex chromosome bivalents. Note the co-localization of the two gene clusters in (c) at two chromosomes. Bar = 5 μm.

**Figure 2:**
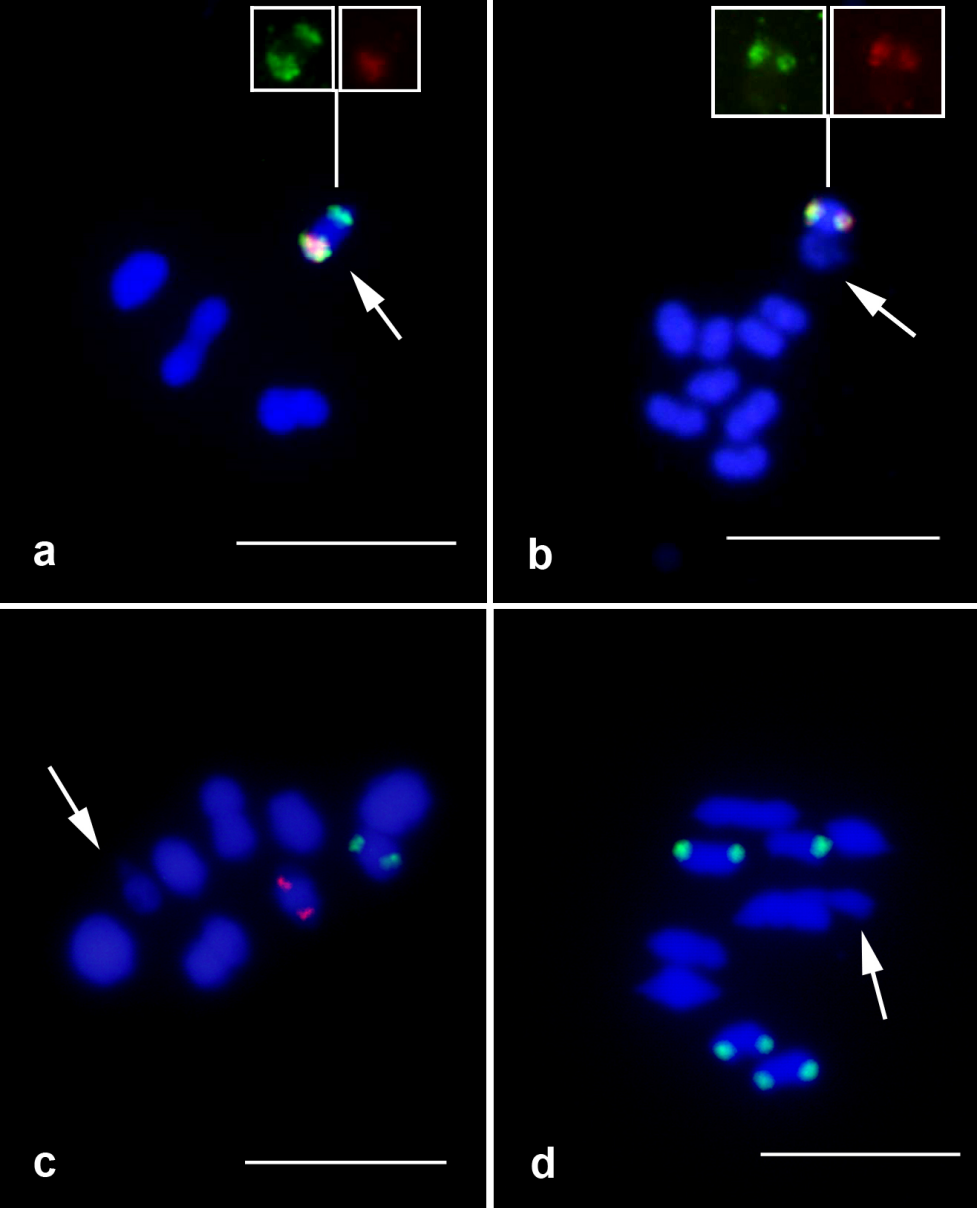
**Cytogenetic mapping of the 5S (red) and 18S (green) rDNA clusters in four species of Scarabaeinae belonging to the (a) Oniticellini, (b) Onthophagini, and (c, d) Coprini tribes**. (a) *Eurysternus caribaeus*, (b) *Digitonthophagus gazella*, (c) *Ontherus apendiculatus *and (d) *O. sulcator*. The arrows indicate the sex bivalents. Note the co-localization of the two gene clusters in (a) and (b). Bar = 5 μm.

**Figure 3:**
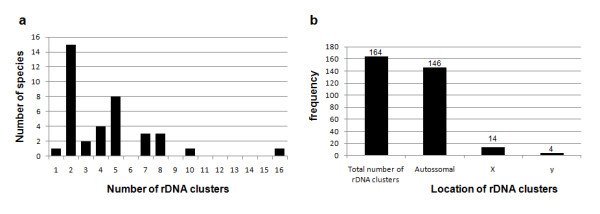
**(a) Distribution of the major rDNA loci among 31 Scarabaeinae species; (b) Total number of major rDNA clusters and their frequency on the autosomal and sex chromosomes**. Species with intraspecific variation were considered twice (see Table 1).

Chromosomal mapping of the 18S and 5S rDNA loci primarily revealed conspicuous clusters on distinct chromosomes (Figures 1, 2). The most common pattern for the 18S rDNA clusters was the presence of two sites (one bivalent). However, the number of clusters for this repeated gene ranged from one to 16, with an average of 4.2 sites per diploid genome, that were located on the autosomes, sex chromosomes or both. The autosomes were the most common location (~89.0% of the sites) (Figures 1, 2, 3 and Table 1). For the 5S rRNA gene almost all species presented only two sites (one bivalent) that were located on one autosomal pair (Figures 1, 2 and Table 1). Distinct 5S rDNA location patterns were observed for several species. In *Eurysternus caribaeus *(Figure 2a), the 5S rDNA was restricted to the × chromosome; in *Diabroctis mimas *(Figure 1c), six clusters were observed (five on the autosomes and one on the × chromosome). In *Coprophanaeus ensifer *(Figure 1a), the clusters were located on the sex chromosomes. In general, the two rRNA genes presented distinct location, although in three species the sites of the 18S and 5S rDNA loci occurred in the same chromosomal region (Figures 1, 2). In *D. mimas*, the sites of the 18S and 5S rDNA loci co-occurred on one autosomal bivalent pair and on the × chromosome (Figure 1c). For *Digitonthophagus gazella *and *E. caribaeus*, these genes were collocated on one autosomal bivalent or on the × chromosome, respectively (Figure 2a, b).

**Figure 4:**
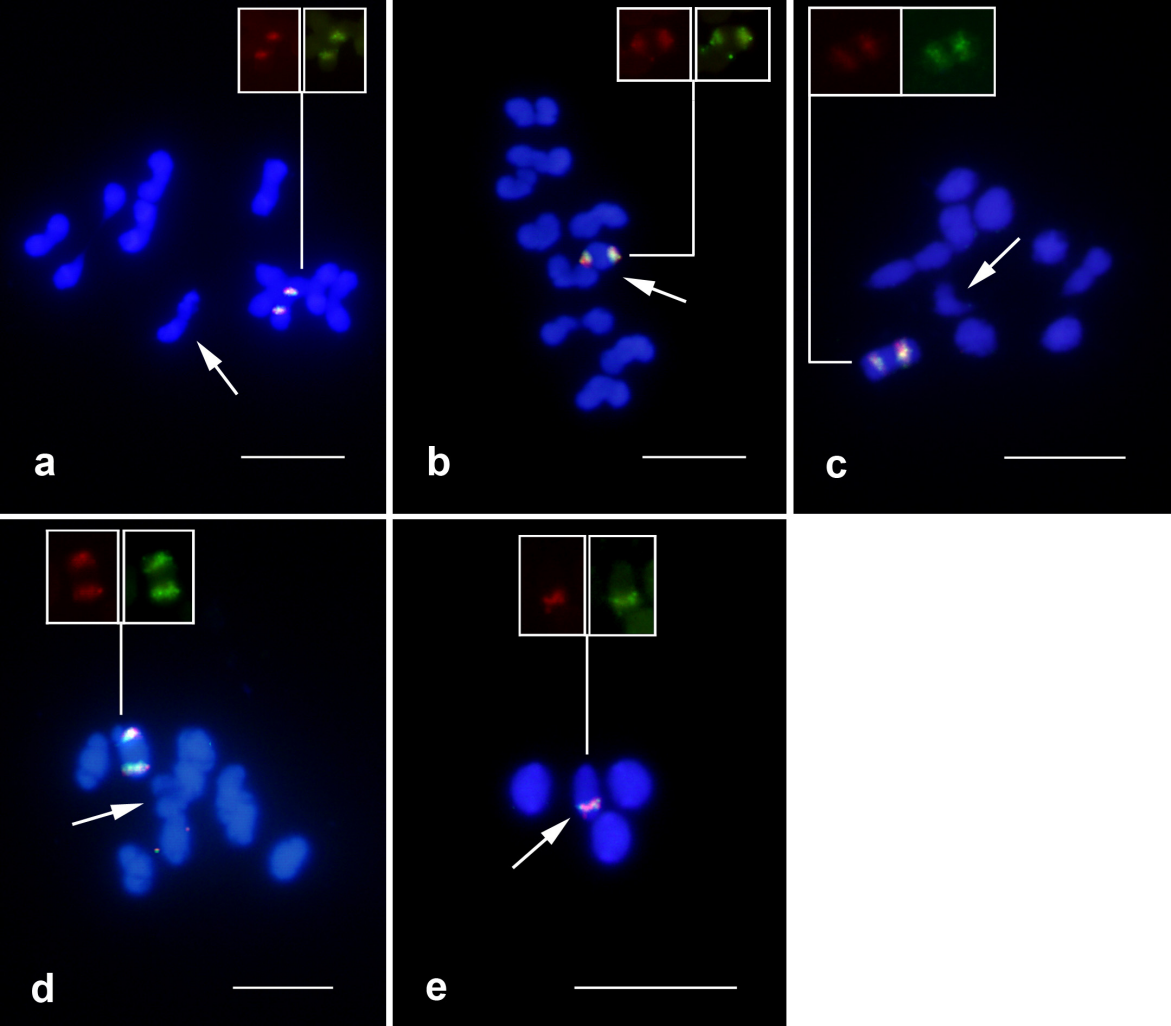
**FISH for the 5S rRNA and histone H3 genes in five Scarabaeinae representatives**. (a) *Coprophanaeus cyanescens*, (b) *C. ensifer*, (c) *Phanaeus splendidulus*, (d) *Deltochilum verruciferum *and (e) *Eurysternus caribaeus*. The arrows indicate the sex bivalents. Note the co-localization of the two clusters in all cells, and the presence of only one cluster for the two genes on the × chromosome in (e). Bar = 5 μm.

FISH for the 5S rRNA and histone H3 genes revealed that these two markers show overlapping signals in the karyotypes of six species randomly selected and in all other previously studied species, indicating that the two genes co-locate (Figure 4, Table 1). In most species, these two sequences were located only on one autosomal bivalent (Figure 4, Table 1). However, in *C. ensifer*, the 5S rDNA and histone H3 gene elements were located on the × and Y chromosomes (Figure 4b), while in *E. caribaeus*, the H3 gene elements were exclusively found on the × chromosome (Figure 4e). In *D. mimas*, these two loci were located on five autosomal chromosomes and the × chromosome (results not shown).

The analysis of interphasic nuclei and early meiotic cells (with less condensed chromosomes/chromatin) hybridized with the 18S and 5S rDNA and histone H3 probes revealed that the 5S rRNA and histone H3 genes overlapped (additional file 1), as observed in the condensed metaphasic chromosomes (Figure 4). However, the 18S rDNA loci were located in a distinct region of the cell from the 5S and histone sites, and only the species that showed co-location of the 5S/18S rDNAs (*E. caribaeus *and *D. gazella*) (Figure 2a, b), showed overlapping signals (additional file1e, f).

An interesting characteristic for the three multigene families studied was the presence of only one site per chromosome (Figures 1, 2, 4). In addition to the variability observed for the 18S rDNA loci in the species studied here for the first time, we identified polymorphisms regarding the number of sites in species that had previously described, such as in *Deltochilum calcaratum*, *C. ensifer*, *C. cyanescens *and *Diabroctis mimas *(Table 1).

Heteromorphism related to the cytogenetic mapping of the three genes in several species was observed with regard to the size and presence/absence of the clusters in the homologous chromosomes, as in, for example, *Ontherus sulcator *(Figure 2d), *D. mimas *(Figure 1c) and *Phanaeus splendidulus *(Figure 1d). For *E. caribaeus *it was observed variability of 18S rDNA cluster size between × and Y chromosomes, and 5S rRNA/H3 histone genes were restrict to × chromosome. Although we were able to define the number of clusters for the genes mapped, the precise positions along the chromosomes were sometimes difficult to determine due the small size and the high condensation level of the chromosomes. It was also difficult to determine the specific chromosome pair that contained the sequences studied in some species due the karyotypic symmetry.

**Figure 5:**
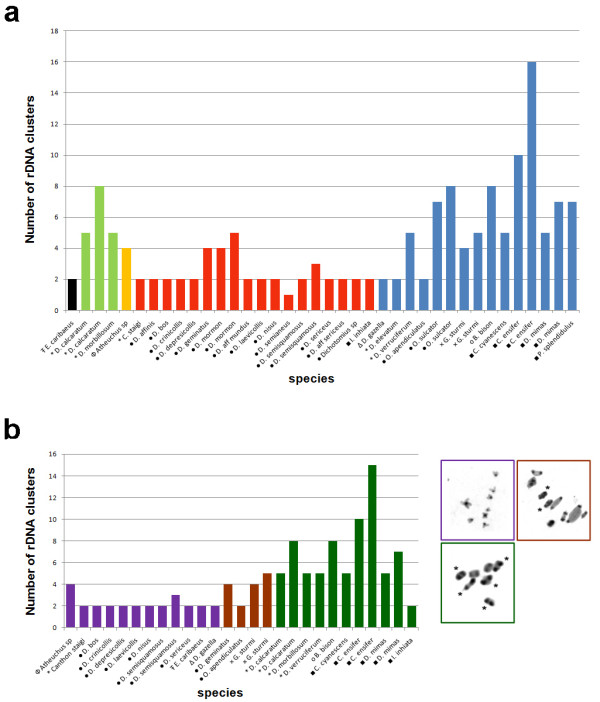
**Comparison of the distribution of the number of 18S rDNA loci and the diploid number in 31 species of Scarabaeinae (a) or with the distribution of heterochromatin in 21 species (b)**. Each symbol indicated below the name of species represents one of the distinct tribes: (T with stroke) Oniticellini, (phi) Ateuchini, (black circle) Coprini, (empty triangle) Onthophagini, (plus sign) Gymnopleurini, (asterisk) Canthonini, (empty circle) Onitini and (black square) Phaneini. Species with intraspecific variation were considered twice (see Table 1). The color of the bars in (a) corresponds to each defined class regarding the diploid number, 2n = 8 (black), 2n = 14 (light green), 2n = 16 (orange), 2n = 18 (red), 2n = 20 (blue), and in (b) the distribution of heterochromatin, pericentromeric blocks (purple), pericentromeric and terminal blocks (brown), pericentromeric blocks and diphasic chromosomes (dark green). In (b), the colored squares correspond to each heterochromatin distribution pattern.

The data obtained regarding the number of major rDNA sites for 31 species (including both the present work and previous published data) were compared to the diploid numbers (2n) and heterochromatin distribution patterns to evaluate the relationship between these karyotype features. The variations in the diploid number and the number of rDNA sites showed no apparent relationship as indicated by a low correlation value (r = 0.21, *P *= 0.199) between the data (Figure 5a). However, a higher number of major rDNA sites were observed in species with increased amounts of heterochromatin (Figure 5b).

## Discussion

### High diversity of the chromosomal distribution of the 18S rDNA clusters and heterochromatin

Considering the major rRNA genes, two main patterns of distribution were detected (i) two rDNA sites (one chromosomal bivalent) harboring these genes, as observed in *Dichotomius*, *Canthon staigi*, *Deltochilum elevatum *and *Ontherus apendiculatus*; (ii) increased numbers of 18S rDNA clusters (ranging from 3 to 16 sites), as observed in *Bubas bison*, *C. ensifer*, *C. cyanescens*, *D. mimas*, *Ontherus sulcator *and three *Deltochilum *species. This suggests that the major rRNA genes are under a distinct evolutionary mechanism regarding cluster spreading.

The two main patterns for the major rDNA distribution were primarily observed for three tribes, Canthonini, Coprini and Phanaeini, which include several species analyzed. Among Coprini species, the clusters of the major rDNA clusters have not suffered intense chromosomal reorganization, as they are primarily associated with only one bivalent, as observed in *Dichotomius *species and *Ontherus apendiiculatus*. Phanaeini is characterized by an intense movement of the major rDNA clusters that resulted in the generation of different numbers of sites on several chromosomes, as observed in *C. ensifer*, which presents the highest number of rDNA clusters (16 sites) within the subfamily, and in Coleoptera [27]. Intraspecific polymorphism with regard to the number of rDNA clusters was observed in *Coprophaneus ensifer*, *C. cyanescens*, and *D. mimas*. In the Canthonini tribe, variable patterns of rDNA clusters were observed, with species presenting with either no spreading of the major rDNA clusters, such as *Canthon staigi *and *Deltochilum elevatum*, or with scattered rDNA clusters, as observed for three of the *Deltochilum *representatives. These results indicate that the major rDNA clusters' ability to move is independent of taxonomic units and may be related to the heterochromatin dispersion (see discussion below).

The ancient condition in Scarabaeinae appears to be the occurrence of one autosomal bivalent as a nucleolar organizer. This theory is corroborated by the presence of the pattern in a large number of species within the group and the sister groups of the subfamily. That distribution pattern is also the most common pattern for Coleoptera as a whole, at least for representatives of Polyphaga [29]. In addition to this common pattern that consists of only one chromosomal pair of major rDNA clusters, an intense repositioning of the major rDNA clusters in Scarabaeinae was involved in the increasing number of rDNA sites and the movement to different autosomes and sex chromosomes. The presence of major rDNA clusters associated with the sex chromosomes in different species could be related to either (i) the occurrence of large chromosomal rearrangements, such as fusions, as observed in *Deltochilum calcaratum*, *D. morbillosum *and *E. caribaeus*, species that have a derived neo-XY sex system, or (ii) the occurrence of transpositions, as observed in *Coprophanaeus*, *D. mimas *and *Deltochilum verruciferum*, which are species with the ancient Scarabaeinae diploid number (2n = 20). Although the occurrence of chromosomal fusions were proposed in some species with a reduced diploid number, the presence of rDNA clusters on the sex chromosomes could also be a result of transpositions if fusion involving only autosomes is considered, as in *Dichotomius *[28].

Although the variation in the number of major rDNA clusters can be attributed to chromosomal rearrangements in some species, there is no correlation between the variation in the rDNA sites and the diploid number. There are examples of species that have a reduction in the diploid number without a modification to the number of rDNA sites, while species with conservation of the ancestral diploid number and extensive repositioning and expansion of major rDNA clusters number have also been identified. There is evidence of the "movement" and "multiplication" of the major rDNA clusters without fusions or other chromosomal rearrangements [30]. In Scarabaeinae, these modifications could be attributed to an ectopic recombination and transposition and to inversions and translocations within the genome. Similar mechanisms are responsible for intra- and interspecific variations in other insects, such as Acrididae grasshoppers [8] and in Lepidoptera [11]. These results indicate distinct evolutionary trends that are related to the macro-chromosomal structure (diploid number, chromosome morphology and sex chromosomes) and the organization of the major rDNA genes in some insect genomes.

The analysis of heterochromatin and major rDNA dispersion revealed an interesting relationship pattern. Species with heterochromatin restricted to the centromeric/pericentromeric regions were primarily characterized as having a stable number of major rDNA that were restricted to one chromosomal bivalent. Only *Ateuchus *sp. had four clusters, while the presence of three clusters in *D. semisquamosus *was a polymorphic condition. However, extensive variability in the number of major rDNA sites was observed in the majority of representatives (except for *Isocopris inhiata*) in which heterochromatin was dispersed and occurred in large quantities within the karyotypes, e.g., large paracentromeric heterochromatic blocks and diphasic chromosomes. In species that showed a moderate dispersion of heterochromatin, the major rDNA clusters spread in two species and was restricted to one autosomal bivalent in another, *Ontherus appendiculatus*. Interestingly in species whose the relationship in position for heterochromatic blocks and major rDNA was possible to determine it was observed a general pattern for non co-localization in some representatives without dispersion for these two chromosomal markers, such as in *Dichotomius *[28]. In species with spreading of these elements in general they were co-located, such as in *Deltochilum *and *Coprophaneus *[26,27]. Our results indicate that the same evolutionary forces might be acting on these two components of the Scarabaeinae genome, resulting in the spreading of the major rDNA clusters along with heterochromatin. This hypothesized pattern of evolution might be favored by ectopic paring during chromocenter formation during the initial meiotic stage. Ectopic pairing is a common behavior in this insect group that appears to play an important role in nucleolar organization and chromosomal segregation [31,32].

The restriction or spreading of the number of rDNA clusters might be associated with the presence or absence of an appropriate molecular mechanism associated with heterochromatin and involved in the ectopic recombination possibly caused by repeated DNAs. The ability of rDNA clusters to move and vary in number was first observed by Schubert (1984) [33] in *Allium*. Since then, some additional evidence has accumulated concerning the ability of rDNA to move within the genome. Recent studies have proposed that transposable elements are a potential source for the movement of rDNA [34,35] and other genes [36,37] to different regions of the genome.

### The conservation of the 5S rRNA and histone H3 genes in Scarabaeinae karyotypes

In contrast to the variability in the number of major rDNA clusters, a high conservation in the number of 5S rRNA and histone H3 gene clusters was observed. For invertebrates, the mapping of these sequences was previously restricted to few species of mollusks, insects, crustaceans, annelids and echinoderms [9,10,12,28,38-42]. In insects, these types of studies have been mainly focused on grasshoppers [9,10,12,42], and only 14 species of beetles had been previously studied, all of which belong to the genus *Dichotomius *[28]. The co-localized clusters (one bivalent) for these two genes in some Scarabaeinae species could indicate that this is the ancient organization for these sequences, and they have not extensively changed in number since the origin of Scarabaeinae [43], despite the diversification of the species. An intense conservation of the number of histone gene clusters, with only one or two chromosomes containing clusters, has also been described in grasshoppers [9,10], mollusks [40,41] and fish species [44,45], although variability has also been reported in these groups. These results might indicate that a strong purifying selection acts on the histone clusters, preventing the spread of these genes through the genome, as was proposed for the grasshoppers [9].

The 5S rDNA gene is highly conserved in Scarabaeinae representatives, and all species examined showed an overlap between the 5S rDNA and the histone H3 genes' signal at the same chromosomal region. This overlap was corroborated by the observation of overlapped signals in cells that were in the initial stages of meiosis and that had interphasic nuclei containing less condensed chromosomes. This indicates that these two multigene families could have a linked organization in the Scarabaeinae genomes. The associated dispersion of the 5S rRNA/histone H3 genes in *D. mimas *and the restriction of these two sequences to the × chromosome of *E. caribaeus*, likely due to unequal cross-over events between the × and Y chromosome in this species [24], reinforces the hypothesis that the 5S rDNA/histone H3 gene clusters are associated in the genome. Additional molecular studies are necessary to fully confirm this hypothesis. An associated or co-localized organization has also been described in mollusks [46], crustaceans [47-49], *Dichotomius *coleopterans [28] and Proscopiidae grasshoppers [10]. Our results reinforce the idea that the association of the 5S rDNA and histone H3 clusters is not sporadic in coleopterans and that it appears to be common. Besides the association of 5S rDNA and histone H3 genes, co-localization or linked organization of major rDNA and histone genes were also reported in insect as described recently for example in *Diuraphis noxia *(Hemiptera) [50], *Anthonomus grandis *and *A. texanus *(Coleoptera) [51].

Unlike the results observed among the representatives of Scarabaeinae, the 5S rDNA cluster is highly dynamic among chromosomes and the genomic dynamism in some animal groups, such as in fish and Acrididae grasshoppers [12,52,53]. This stability in Scarabaeidae beetles could be the result of its association with the histone genes, which may result in the same purifying selection that appears to act against the spread of histone clusters.

In contrast to the co-localization of the 5S rDNA/histone H3 clusters, the 18S rDNA is not co-localized in the genomes of the Scarabaeinae species studied. Only *Diabroctis mimas *and *Digitonthophagus gazella *showed a co-localization of these sequences. These results could be explained by a transposition of the 18S rDNA cluster due to its intense movement in the genome of some species. This physical separation could be result in a functional advantage for these ribosomal sequences. The disassociation of the two multigene families that encode for rRNAs is a common pattern for eukaryotic and vertebrate chromosomes, including those in fishes [54-56]. However, some invertebrate species have co-localized rRNA clusters, including representatives of the annelids, mollusks and crustaceans; however, a non co-localized organization has also been described [38,48,57,58].

The association/co-localization of multigene families in animal genomes has been reported for some sequences, including rRNAs, the histone genes and small nuclear RNAs (snRNAs). These associations/co-localizations are poorly understood, and their biological effect is still unclear. According to studies by Dover (1986) [59] and Liu and Fredga (1999) [60], the linkage is important to maintain multiple, conserved arrays. Kaplan et al. (1993) [61] hypothesized that the association of the repetitive multigene families might play a functional role in the organization of the nucleus. In the case of the 18S rDNA, 5S rDNA and histone H3 histone, the separation of the 18S and 5S rDNA arrays might convey a functional advantage, since the 18S rRNA gene is transcribed by RNA polymerase I and the 5S rRNA gene is transcribed by RNA polymerase III. However, the association of the histone H3 and 5S rRNA genes cannot be explained by a transcriptional advantage because these two sequences are transcribed by different polymerases.

## Conclusions

The high variability in the karyotype organization previously observed in Scarabaeinae representatives resulted from distinct chromosome rearrangements during their evolution [18] is also observed for heterochromatin and the 18S rDNA clusters. Our results indicate that these two genomic elements (18S rDNA and heterochromatin) likely are subjected to similar evolutionary forces that regulate their spreading in the distinct Scarabaeinae subfamily lineages. The conservation of the location and number of the 5S rRNA and histone H3 gene clusters indicates that these multigene families are likely under the control of different evolutionary forces than the 18S rDNA clusters and heterochromatin. This separation reinforces the idea that evolutionary spreading mechanism might operate differently at multigene families and other repeat elements.

## Methods

Samples from adult males of 13 species of Scarabaeinae beetles were collected in distinct cities in the Minas Gerais, Paraná, Pernambuco, and São Paulo states in Brazil. The testes were fixed in Carnoy (3:1 ethanol:acetic acid) and were stored in the freezer at -20°C. To check the male karyotypes, the slides were stained with 2% lacto-acetic orcein. The chromosome preparations for the C-banding and fluorescence in situ hybridization (FISH) experiments were made by squashing using a drop of 45% acetic acid and subsequently removing the coverslip after immersion in liquid nitrogen. The C-banding experiments were performed according the protocol described by Sumner (1972) [62].

DNA probes for 5S rDNA and H3 histone sequences were obtained from cloned fragments obtained from the genome of the beetle *Dichotomius semisquamosus *[63]. The 18S rRNA and histone H3 gene probes were labeled by nick translation using biotin-11-dATP (Invitrogen, San Diego, CA, USA), and the 5S rRNA gene was labeled with digoxigenin-11-dUTP (Roche, Mannheim, Germany). The FISH procedures were performed according to the method adapted by Cabral-de-Mello et al. (2010b) [63]. Preparations were counterstained with 4,6-diamidino-2-phenylindole (DAPI) and mounted in Vectashield (Vector, Burlingame, CA, USA). Images were captured using an Olympus DP71 digital camera coupled to a BX61 Olympus microscope and were optimized for brightness and contrast using Adobe Photoshop CS2.

Statistical analysis was performed using the Pearson rank test to analyze the degree of correlation between the number of 45S rDNA sites and the diploid number. A comparative analysis between the distribution of heterochromatin and the number of 45S rDNA sites was also performed.

## List of abbreviations

2n: diploid number; DAPI: 4, 6-diamidino-2-phenylindole; FISH: fluorescence *in situ *hybridization; rDNA: ribosomal DNA; rRNA: ribosomal RNA; snRNA: small nuclear RNA

## Competing interests

The authors declare that they have no competing interests.

## Authors' contributions

DCCM, CM and RCM designated and coordinated the study and drafted and revised the manuscript. DCCM and SGO carried out the experimental analysis. All authors read and approved the final manuscript.

## Supplementary Material

Additional file 1 **Fluorescence *in situ *hybridization using as probes 18S and 5S rDNAs and histone H3 in initial meiotic and interphasic nucleus of Scarabaeinae beetles species**. Initial meiotic cells (a-e, h, i) and interphasic nuclei (f, g) hybridized with probes for the 18S (green) and 5S rDNA (red) genes (a-f) and the histone H3 (green) and 5S rRNA (red) genes (g-i). (a) *Deltochilum elevatum*, (b) *Deltochilum calcaratum*, (c) *Dichotomius crinicollis*, (d) *Coprophanaeus cyanescens*, (e, f) *Diabroctis mimas*, (g) *Dichotomius bos*, (h) *Dichotomius laevicollis *and (i) *Deltochilum verruciferum*. Note the separation of the 18S and 5S rDNA signals in (a-e). In (f), two small signals for 18S and 5S rDNA overlap, and note the overlapped configuration of 5S rRNA and histone H3 genes in (g-i). Also note the formation of the chromocenter by heterochromatic sequences (a, b, d, e and i). A scale bar is not shown. Click here for file
