# Supplementary figures and images for: Chromosomal organization of the 18S and 5S rRNAs and histone H3 genes in Scarabaeinae coleopterans: insights into the evolutionary dynamics of multigene families and heterochromatin
Source: BMC Genet. 2011 Oct 15;12:88. doi: 10.1186/1471-2156-12-88 (PMC3209441; doi:10.1186/1471-2156-12-88)

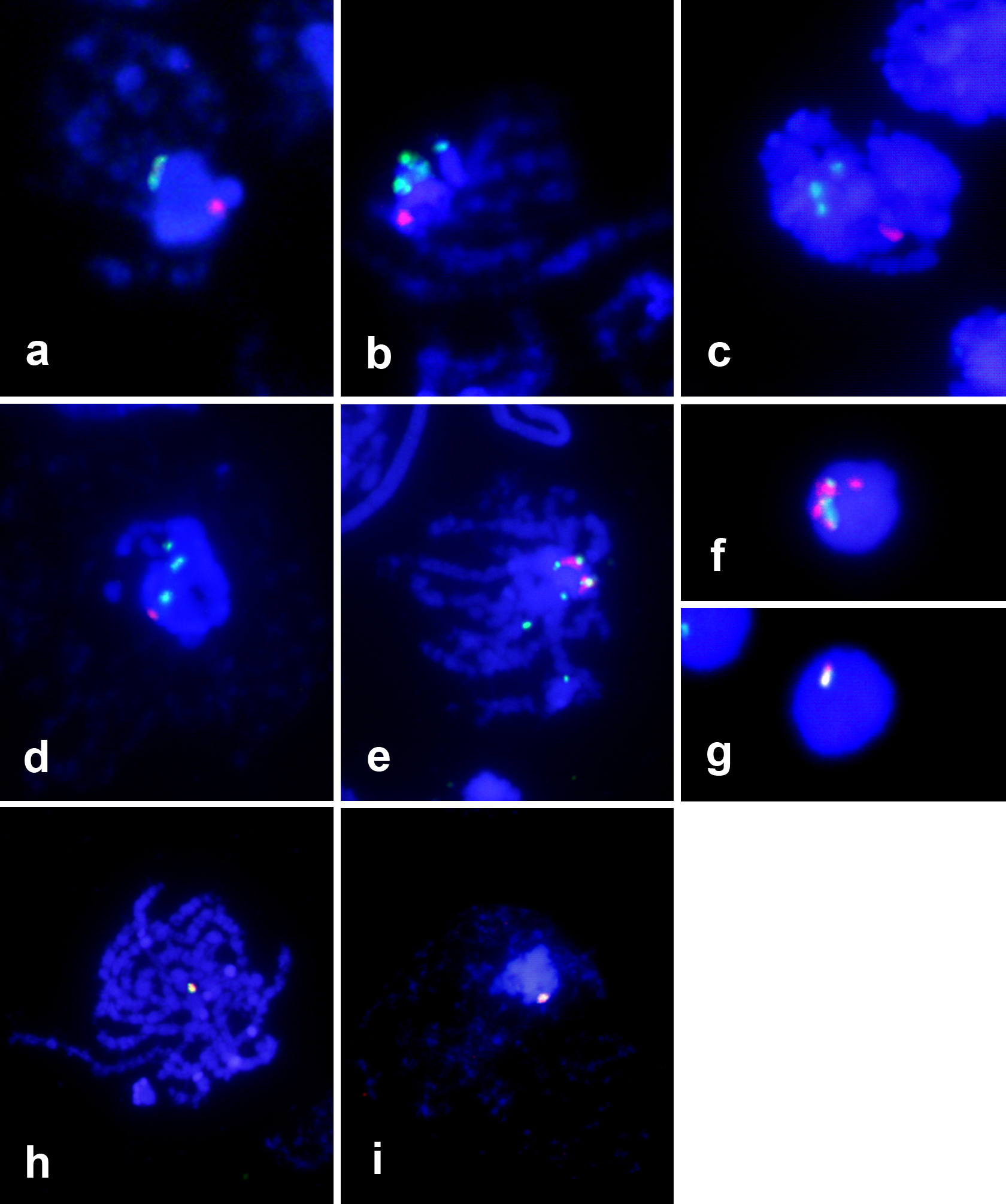

Supplement: Additional file 1 — Fluorescence in situ hybridization using as probes 18S and 5S rDNAs and histone H3 in initial meiotic and interphasic nucleus of Scarabaeinae beetles species. Initial meiotic cells (a-e, h, i) and interphasic nuclei (f, g) hybridized with probes for the 18S (green) and 5S rDNA (red) genes (a-f) and the histone H3 (green) and 5S rRNA (red) genes (g-i). (a) Deltochilum elevatum, (b) Deltochilum calcaratum, (c) Dichotomius crinicollis, (d) Coprophanaeus cyanescens, (e, f) Diabroctis mimas, (g) Dichotomius bos, (h) Dichotomius laevicollis and (i) Deltochilum verruciferum. Note the separation of the 18S and 5S rDNA signals in (a-e). In (f), two small signals for 18S and 5S rDNA overlap, and note the overlapped configuration of 5S rRNA and histone H3 genes in (g-i). Also note the formation of the chromocenter by heterochromatic sequences (a, b, d, e and i). A scale bar is not shown. [file 1471-2156-12-88-S1.JPEG]
